# Research progress of proteolysis-targeting chimeras in viral hepatitis drug discovery

**DOI:** 10.3389/fchem.2026.1810498

**Published:** 2026-06-12

**Authors:** Xiaofei Hao, Yanli Wang, Gang Huang, Chaoyang Lin, Peng Zhao, Wubin Zhi, Xiankai Li, Peng Qi, Peng Liu

**Affiliations:** 1 School of Chemical Engineering Zhengzhou University, Zhengzhou, China; 2 High and New Technology Research Center, Henan Academy of Science, Zhengzhou, China; 3 Institute of Chemistry of Henan Academy of Sciences, Zhengzhou, China

**Keywords:** antiviral drugs, fluorine, hepatitis virus, PROTAC, resistance

## Abstract

Proteolysis Targeting Chimera (PROTAC) is an innovative drug discovery strategy that utilizes bifunctional small molecules to bring a target protein (POI) into proximity with an E3 ubiquitin ligase, thereby inducing ubiquitination of the pathogenic protein and its subsequent degradation by the proteasome. PROTACs exhibit a novel mechanism of action, catalytic efficiency, high selectivity, and broad applicability. They have achieved significant breakthroughs, particularly in oncology, with several molecules currently in phase II/III clinical trials. The application of PROTAC technology in antiviral drug development remains in the early exploratory stage. However, since most antiviral targets are viral proteins, targeted clearance of these pathogenic proteins is unlikely to cause adverse side effects. PROTACs hold particular promise in chronic viral infections such as hepatitis B virus (HBV), where degradation of proteins like HBx and HBcAg, in combination with existing therapies, may help overcome the challenges of achieving a cure. This article summarizes recent advances in PROTAC-based anti-hepatitis research and critically discusses both the opportunities and challenges in developing PROTACs as next-generation antiviral therapeutics. It also highlights the role and characteristics of fluorine in the design of drugs for treating hepatitis.

## 1 Introduction

Hepatitis viruses, including hepatitis A (HAV), B (HBV), C (HCV), D (HDV), and E (HEV), represent a significant global health burden. Notably, HBV and HCV infections account for the majority of chronic hepatitis cases worldwide, with infected individuals facing substantially increased risks of severe complications such as liver cirrhosis and hepatocellular carcinoma—major causes of liver-related morbidity and mortality (Ligat et al., 2018). These persistent viral infections continue to pose significant challenges to healthcare systems in both developed and developing countries (Ward and Hinman, 2018).

According to the World Health Organization (WHO) 2024 Global Hepatitis Report, there were approximately 254 million cases of hepatitis B virus (HBV) and 50 million cases of hepatitis C virus (HCV) worldwide in 2022, with 2.2 million new infections. Alarmingly, viral hepatitis caused 1.3 million deaths in 2022, with HBV accounting for 83% and HCV for 17% of these deaths, making it the second-leading infectious cause of death globally after COVID-19. Despite a decline in new HBV and HCV cases compared to 2020, mortality rates have increased, underscoring the urgency of global hepatitis control. In response, the 69th World Health Assembly adopted the 2016–2021 Global Health Sector Strategy on Viral Hepatitis, aiming to eliminate viral hepatitis as a public health threat by 2030.

In 2013, Sofosbuvir was approved by the US FDA. It was the first interferon-free direct-acting antiviral (DAA) to achieve sustained virologic response (SVR) rates exceeding 95%, marking the beginning of a new era in which hepatitis C became a curable disease (Wedemeyer et al., 2020). This breakthrough has also intensified research efforts aimed at the elimination of HBV (Inserra et al., 2018).

Current anti-HBV therapies primarily include interferon-α (IFN-α) and nucleos(t)ide analogs (NAs) (Kim, 2018). IFN-α offers high seroconversion rates and low risks of relapse and resistance but requires invasive administration and can cause side effects such as fever, myalgia, and depression (Trépo et al., 2014). The efficacy of IFN-α is also influenced by HBV genotype, with genotype A showing the best response and genotype D the poorest (Kramvis, 2014). Although widely used, NAs are associated with nephrotoxicity, hepatotoxicity, viral rebound upon discontinuation, and high rates of resistance (Buti et al., 2016; Terrault et al., 2015).

Drug resistance remains a significant challenge in antiviral development (Manzoor, 2015). Particularly when a single drug is used over an extended period, resistance can develop rapidly, resulting in treatment failure (Lampertico et al., 2017; Liu et al., 2016; Lok et al., 2015; Park et al., 2016; Terrault et al., 2015). Therefore, novel strategies, such as exploiting alternative mechanisms like targeted protein degradation (TPD), are essential.

Targeted protein degradation (TPD) has demonstrated significant potential in treating serious diseases as an emerging area of drug development (Bondeson et al., 2015). TPD technology can specifically recognize the protein of interest (POI) and cleverly harness the cell’s inherent protein degradation pathways to degrade the POI, thereby avoiding genotoxicity and offering novel approaches for antiviral therapy (Crew et al., 2017; Tinworth et al., 2019).

PROTAC, as a widely utilized key component of TPD technology, operates via a unique “event-driven” mechanism. This mechanism enables effective degradation of the POI without requiring prolonged or high-affinity binding to the target. Additionally, PROTACs retain the advantages of small-molecule drugs, such as high oral bioavailability and strong tissue penetration (Dale et al., 2021). This article provides a comprehensive review of PROTAC’s mechanism of action, technical advantages, and its applications in the treatment of hepatitis viruses.

## 2 Molecular design and mechanism of action of PROTAC

### 2.1 The mechanism of PROTAC degradation

**FIGURE 1:**
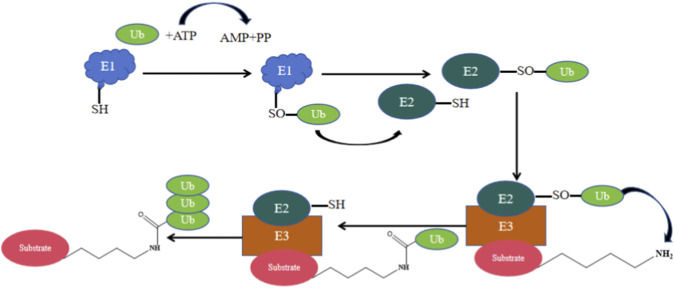
Ubiquitination process.

The mechanism of PROTAC degradation can be summarized into three steps (Figure 1): First, target protein recognition, where the PROTAC molecule precisely binds to POI through its target protein ligand. Next, target protein ubiquitination occurs, wherein the E3 ligase catalyzes the transfer of ubiquitin from the E2 enzyme to the target protein, forming a linkage between the lysine residue of the target protein and the C-terminus of ubiquitin. This process involves multiple repetitive steps, resulting in polyubiquitination of the target protein. Finally, the target protein is degraded, as the ubiquitin-tagged target protein is recognized and degraded by the proteasome system (Pickart, 2001).

Ubiquitin (Ub) is a small protein consisting of 76 amino acids with a molecular weight of approximately 8.5 kDa. At its C-terminus, there is a flexible Gly-Gly dipeptide tail (Gly75–Gly76), and these two glycines serve as the “active fingers” in ubiquitination reactions. The chemical essence of ubiquitination is the condensation between the C-terminus carboxyl group of Gly76 in ubiquitin and the side-chain ε-amino group of a lysine residue on the target protein (Komander and Rape, 2012).

When additional ubiquitin molecules are attached to lysine residues on ubiquitin itself, polyubiquitin chains are formed: K48- and K11-linked polyubiquitination serve as proteasomal degradation signals, whereas K63-linked chains are primarily involved in DNA repair and signal transduction (Lai and Crews, 2016).

Protein ubiquitination is a post-translational modification process involving multiple functional proteins. Its main steps include: 1) Activation, where the C-terminus of ubiquitin forms a thioester bond with a cysteine residue of the E1 ubiquitin-activating enzyme in the presence of ATP; 2) Conjugation, where the activated ubiquitin is transferred from E1 to the E2 ubiquitin-conjugating enzyme; and 3) Ligation, where the E3 ubiquitin ligase catalyzes the final step of the ubiquitination cascade, transferring ubiquitin from E2 to the target protein. This unique mode of action provides PROTACs with inherent advantages, making it a novel drug design strategy (Kazantsev and Krasavin, 2021; Pang et al., 2017; Yau and Rape, 2016).

### 2.2 Molecular design of PROTAC

**FIGURE 2:**
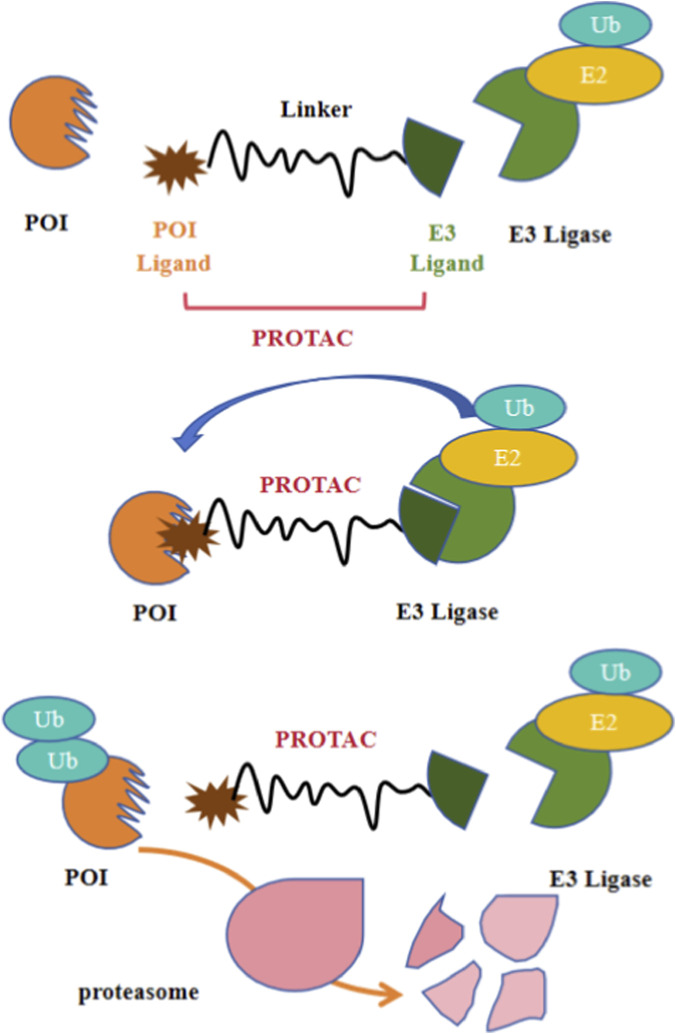
Structure and mechanism of the PROTAC.

From a molecular design perspective, PROTACs are bifunctional molecules composed of three key components: a target protein ligand, a linker, and an E3 ubiquitin ligase ligand (Figure 2). This unique structural design enables PROTACs to simultaneously bind both the target protein and the E3 ligase, forming a stable ternary complex in spatial proximity (Dale et al., 2021; Pettersson and Crews, 2019). Subsequently, the E3 ligase mediates the transfer of ubiquitin from the E2 enzyme to the target protein, marking it with polyubiquitin chains. This ubiquitination signal ultimately directs the target protein for degradation by the proteasome system.

In principle, the efficacy of a PROTAC molecule relies not only on its ability to bring the POI into close proximity with an E3 ubiquitin ligase, but also on enabling the C-terminus COOH of ubiquitin Gly76 to form an isopeptide bond with the ε-NH_2_ of a lysine residue on the POI. Although the human genome encodes more than 600 E3 ubiquitin ligases that collectively ensure selective degradation of tens of thousands of proteins, only a limited subset—most notably VHL, CRBN, and IAP—has thus far been successfully exploited for PROTAC development (Li and Crews, 2022; Yang et al., 2022). Beyond the specific context of hepatitis viruses, recent comprehensive reviews highlight the remarkable evolution of this field. Notably, Ebadi et al. provided an updated architectural atlas of E3 ligases, detailing the discovery of mechanistically distinct classes such as RZ finger​ and RCR ligases, alongside the well-established RING, HECT, and RBR families (Ebadi et al., 2025). Furthermore, the authors emphasized that the diversity of ubiquitin chain topologies—ranging from canonical K48-linked degradation signals to branched and non-degradative chains—adds layers of regulatory complexity to degrader design. This expanding understanding of E3 biology and ubiquitin signaling provides the foundational framework for developing next-generation PROTACs capable of addressing currently undruggable targets. On the POI side, ligands may be derived from antibodies, protein binding domains, or small molecules. The affinity and selectivity of both ligands are essential for efficient ternary complex formation, while the linker properties—including length, conformation, and stability—are equally critical. Excessive linker rigidity may hinder the accommodation of the protein surface topology, whereas excessive flexibility can destabilize the ternary complex, thereby reducing degradation efficiency.

### 2.3 The development history of PROTAC technology

PROTACs were first developed in 2001 as peptide-based molecules, primarily serving as chemical protein knockout tools for *in vitro* validation and mechanistic studies (Sakamoto et al., 2001). Although valuable as biochemical tools, their therapeutic application was limited due to challenges such as poor cell permeability and drugability. Nevertheless, this did not dampen researchers’ enthusiasm for PROTAC development. Given the greater potential of non-peptidic PROTACs for drug discovery, numerous small-molecule PROTACs targeting various proteins have been continuously developed over the subsequent 2 decades (Li et al., 2022; Wang et al., 2022; Wu et al., 2021; Xiang et al., 2021).

Currently, PROTAC molecules have made significant progress in research areas including cancer, inflammatory diseases, and neurological disorders, with several candidates advancing to Phase III clinical trials. However, their application in antiviral therapy, particularly against hepatitis viruses, remains relatively underexplored. In the following sections, we will review the progress of PROTAC technology in the development of antiviral drugs for the treatment of viral hepatitis.

## 3 The application of PROTAC in hepatitis virus drugs

The rationale for deploying PROTACs against Hepatitis B (HBV) and C (HCV) viruses is rooted in the unique ‘Achilles’ heel’ of these pathogens: their absolute dependence on the host ubiquitin-proteasome system (UPS)​ for the entire viral lifecycle.

In the context of HBV, the virus does not merely utilize the UPS; it actively rewires it to establish a pro-oncogenic niche. As reviewed by Kar et al. the HBV-encoded X protein (HBx) functions as a molecular hijacker of the CRL4-DDB1 E3 ligase complex. HBx recruits host E3 ligases to degrade critical tumor suppressors (e.g., pVHL, SMC5/6) and stabilizes oncoproteins (e.g., β-catenin), thereby creating a cellular environment conducive to both viral persistence and hepatocarcinogenesis. This pathological state is sustained by a dramatically altered ubiquitination-dependent proteostasis (Kar et al., 2024).

Similarly, HCV and related Flaviviridae members exhibit a ‘pan-ubiquitination’ dependency. According to Cai et al., HCV exploits ubiquitination and ubiquitin-like modifications (SUMO/ISGylation) at every discrete stage: from TRIM7-mediated K63-linked ubiquitination of the E protein for viral entry, to K27-linked ubiquitination of NS5B by TRIM26 to enhance replication complex formation, and finally to March8-mediated ubiquitination of NS2 for viral egress (Cai et al., 2022).

Given that both viruses rely so heavily on the host’s enzymatic machinery for their propagation, targeting the viral proteins directly often leads to resistance due to high mutation rates. PROTAC technology offers a paradigm shift: by leveraging this very same ubiquitination machinery that the viruses depend upon, PROTACs can enforce the degradation of ‘undruggable’ viral factors (like HBx) or pathogenic host co-factors, effectively stripping the virus of its protective shield and eliminating reservoirs that traditional antivirals cannot reach.

### 3.1 PROTAC against HBV

The Hepatitis B virus (HBV) genome is small and compact (∼3.2 kb, partially double-stranded DNA), encoding seven proteins, including HBcAg, HBeAg, HBx, HBV polymerase, and three forms of HBsAg, from four overlapping open reading frames. HBx is a regulatory protein involved in HBV replication and modulation of host cells. HBcAg is the core antigen that forms the viral capsid, providing protection. HBV polymerase is a multifunctional enzyme composed of four domains: terminal protein, spacer, reverse transcriptase, and RNase H. All these viral proteins are essential for the HBV life cycle, and selectively degrading them represents a promising strategy to suppress viral replication.

**FIGURE 3:**
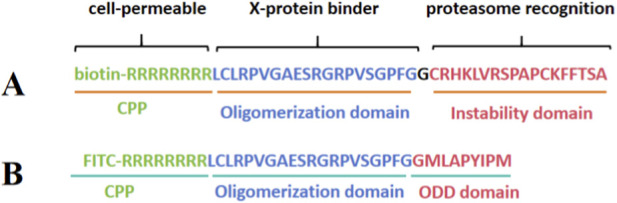
Sequences of the X-protein targeting PROTACs based on the **(A)** instability domain of the X-protein, and **(B)** ODD domain of HIF-1a.

The X protein of HBV (HBx) functions as a “switch” for viral replication, activates transcription of the covalently closed circular DNA (cccDNA), and is a major risk factor for the development of HBV-induced hepatocellular carcinoma (HCC). HBx promotes cancer progression by interfering with host immune responses and apoptotic pathways. Due to its small size and lack of enzymatic activity, HBx has traditionally been considered a challenging drug target; however, targeted protein degradation (TPD) offers new therapeutic opportunities. In 2014, Kristopher Montrose et al. pioneered the first anti-HBV PROTAC degrader (Montrose and Krissansen, 2014). They engineered this novel molecule by fusing the N-terminal oligomerization domain and the C-terminal unstable domain of HBx, while incorporating a polyarginine cell-penetrating peptide (CPP) to facilitate cellular uptake. Through binding assays focusing on the oligomerization domain (amino acids 1–50), they identified residues 16–35 as the minimal and optimal ligand for HBx dimerization. The C-terminal region of HBx (amino acids 140–154) is considered a potential degron because it is an unstable region containing E3 ligase recognition signals and is rich in lysine residues. This PROTAC effectively mediates HBx degradation in cell culture by concurrently engaging HBx and recruiting an E3 ubiquitin ligase (Figure 3).

HBx is a critical regulator of HBV replication and chronic infection, influencing cccDNA formation as well as host DNA repair, transcription, and epigenetic modifications. As an “undruggable” target, HBx is challenging to inhibit using traditional small-molecule approaches, making its targeted degradation a promising strategy for achieving a functional cure of HBV. Several E3 ligases, including TRIM family proteins (e.g., TRIM21, TRIM25, TRIM26, TRIM28) and Siah-1 and Siah-2, have been shown to promote HBx degradation (Hu et al., 2024; Jeong et al., 2019; Song et al., 2021; Yeom et al., 2017; Zhao et al., 2011). HBx-PROTACs have demonstrated potential in cellular and some animal studies, significantly reducing HBV replication and surface antigen levels. However, they have not yet entered clinical trials, primarily due to challenges in HBx ligand discovery, delivery, and pharmacokinetic optimization. Future research directions include the structure-based design of small-molecule HBx ligands and the development of liver-targeted delivery systems for peptide-based PROTACs (e.g., liposomes and nanoparticles).

**FIGURE 4:**
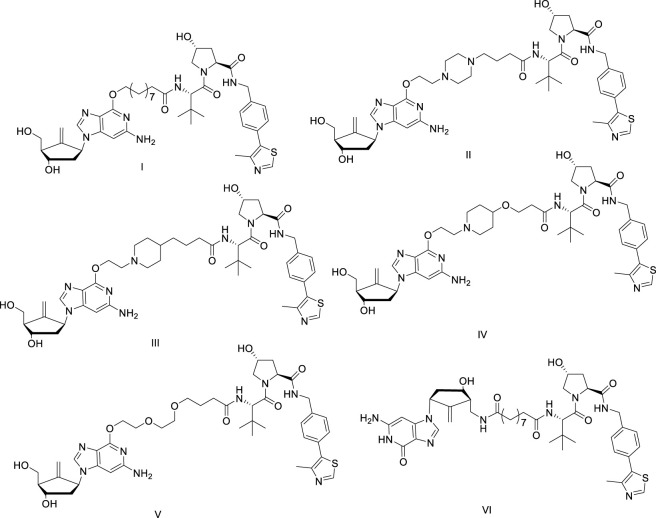
Chemical structures of LGP-LK-LGE.

Patent WO2022156764A1 describes a class of small-molecule compounds based on PROTAC technology (Su et al., 2022). As shown in Figure 4 the core structure comprises three components: a ligand targeting viral DNA polymerase (LGP), an E3 ubiquitin ligase ligand (LGE), and a linker (LK) connecting the two. The LGP is selected from nucleoside antivirals, such as entecavir. The LGE utilizes a VHL ligand to recruit the E3 ubiquitin ligase complex. The linker consists of flexible alkyl chains or polyethylene glycol segments, which regulate the molecular spatial conformation through amide and ether bonds to ensure efficient formation of the ternary complex (viral polymerase-PROTAC-E3 ligase).

**FIGURE 5:**
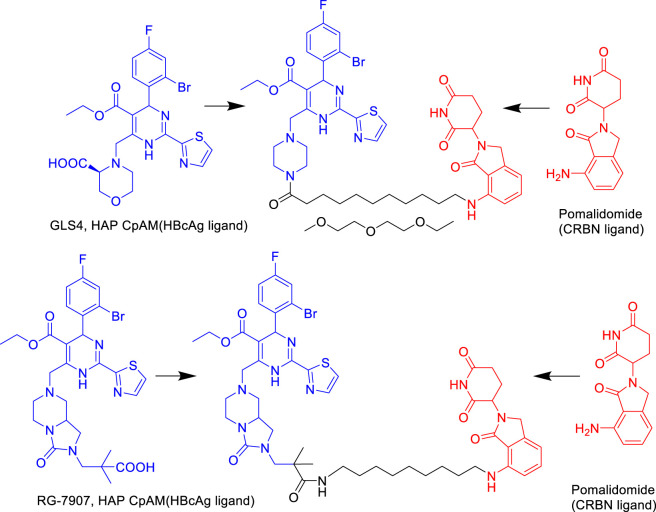
Chemical structures of HAP CpAM and HAP-PROTAC.

Experimental data demonstrate that in HBV-infected HepG2.2.15 and HepAD38 cell models, the representative compound TPD00203 (Compound I, Figure 5) significantly inhibited viral DNA replication (>60% inhibition, p < 0.001) after 7 days of treatment at a low concentration of 10 nM. At 10 μM, it achieved more than 50% degradation of viral polymerase within 36 h (quantified by Western blot). This degradation was completely reversed by the proteasome inhibitor MG132, confirming its dependence on the ubiquitin-proteasome pathway. Further NanoBiT experiments using the engineered Huh7-HBP reporter cell line revealed that TPD00203 reduced intracellular polymerase levels to 48% of the control group after 48 h of treatment at 300 nM.

Nucleoside antivirals typically require kinase-mediated phosphorylation to form triphosphates before they can inhibit viral polymerase function. Therefore, entecavir essentially acts only as a ligand for nucleoside kinases, while TDP00203 might indirectly eliminate HBV polymerase by degrading nucleoside kinases. Given the high efficacy of nucleoside analogs in suppressing HBV reverse transcriptase, the prospects for PROTACs targeting HBV polymerase appear limited.

The HBV core protein (HBcAg or Cp) consists of 183 or 185 amino acids and is divided into two major regions: the N-terminal region (amino acids 1–149), which is highly conserved and responsible for self-assembly into an icosahedral capsid; and the C-terminal region (amino acids 150–183/185), which is highly flexible and rich in lysine and arginine residues containing ε-NH_2_ groups, functioning in pgRNA encapsidation. HBcAg forms dimers as the basic structural units, which further assemble into icosahedral capsids approximately 34–36 nm in diameter. The α-helix bundles at the dimer core serve as small-molecule binding pockets that can bind capsid inhibitors with high affinity, inducing aberrant capsid assembly. Because HBcAg possesses well-defined small-molecule binding pockets and potential ubiquitination sites, it represents a rational target for PROTAC technology. By utilizing existing capsid assembly inhibitors (such as GLS4, RG-7907, and GLP-26) as POI ligands and linking them to suitable E3 ligase ligands (CRBN or VHL), it may be possible to induce the degradation of HBcAg.

**FIGURE 6:**
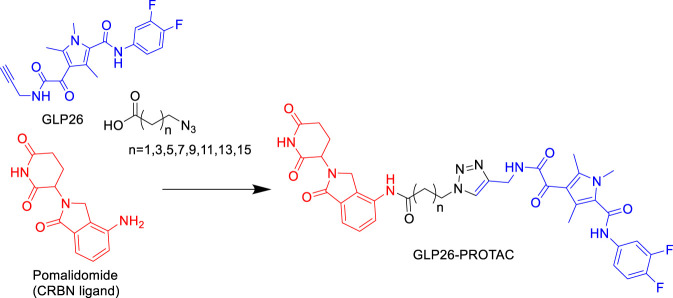
Chemical structures of BSA CpAM and GLP26-PROTAC.

In 2021, Ma (2021), Liu et al. (2021) reported the inhibitory effects of dual-ligand PROTACs derived from two heteroaryldihydropyrimidine (HAP) capsid inhibitors, GLS4 and RG-7907 (Figure 6), respectively. In cellular assays, All these HAP-PROTAC analogs demonstrated significant inhibition of HBV DNA replication, exhibiting submicromolar EC50 values comparable to those of their parent compounds. Toxicity tests showed that the introduction of E3 ligase ligands reduced cytotoxicity, and the physicochemical properties of the linker significantly influenced safety profiles. Mechanistic studies further revealed fundamental differences between GLS4-PROTAC analogs and the parent molecule GLS4: while GLS4 primarily acts by inducing capsid misassembly, the PROTAC analogs drastically reduced total capsid levels and degraded core proteins via the ubiquitin-proteasome pathway, with some compounds reducing core protein levels to below 10%. These findings not only highlight the potential of PROTAC technology in antiviral drug development but also provide critical data for optimizing molecular design (e.g., linker length, hydrophilicity, and ligand selection), thereby laying a foundation for next-generation HBV therapies with enhanced efficacy and reduced toxicity as shown in Table 1.

**TABLE 1:** Anti-HBV activity of compounds GLP26-PROTAC.

n	1	3	5	7	9	11	13	15
EC_50_ (μM)	0.5	4.5	2.2	34.8	63.9	3.23	1.6	0.019

**FIGURE 7:**
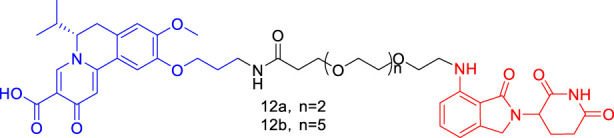
Chemical structures of 12a and 12b.

From a mechanistic perspective, HBV core protein allosteric modulators (CpAMs) can be divided into two categories. In addition to the aforementioned HAPs, which induce misassembly of core proteins resulting in aberrant capsids, there are also sulfamoylbenzamide (SBA) derivatives that interfere with pregenomic RNA (pgRNA) encapsidation, leading to the formation of empty capsids (Zhou et al., 2017). Our group investigated a bifunctional PROTAC molecule based on the second class of CpAMs (Figure 7). Using GLP26 as the HBV core protein ligand and linking it to a CRBN ligand, we synthesized and evaluated a series of GLP26-PROTACs for anti-HBV activity (Hao et al., 2025). We found that the length of the linker is critical for its *in vitro* bioactivity.

The crystal structure of the HBV core protein has been extensively characterized. It features a highly conserved N-terminal region essential for HBV capsid assembly, as well as a C-terminal intrinsically disordered region enriched with lysine and arginine residues, which facilitates ubiquitination. Numerous small-molecule ligands, known as CpAMs, have been developed, with some advancing to phase III clinical trials. By systematically screening combinations of these CpAMs with E3 ubiquitin ligase ligands and linkers, it is highly promising to achieve efficient targeted degradation of the core protein using PROTAC technology.

**FIGURE 8:**
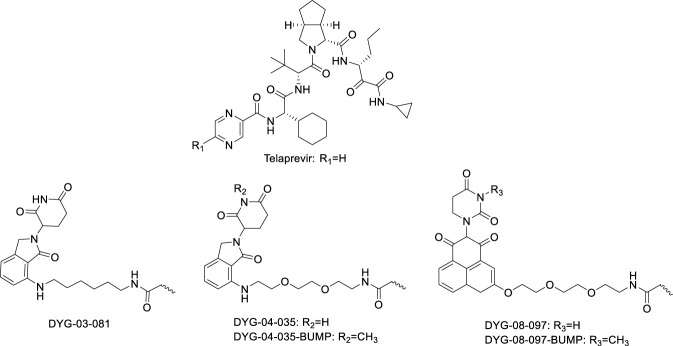
Chemical structures of telaprevir and DGY-03-081, DGY-04-035, DGY-08-097.

In 2024, Li You and colleagues reported a series of dihydroquinolizinone (DHQ)-based PROTAC compounds (Li, et al., 2024) designed for the targeted degradation of HBsAg. The PROTAC molecules (12a and 12b, Figure 8) were created by conjugating an E3 ubiquitin ligase ligand to the HBsAg inhibitor RG7834 via a PEG linker. Antiviral efficacy was assessed using a nanoluciferase-tagged HAV reporter virus (HAV18f-Nluc) and HBV-replicating cell lines (HepG2.2.15 and Huh7). Both compounds demonstrated significant inhibition of HAV replication, with IC50 values of 576 nM (12a) and 277 nM (12b), while exhibiting weaker activity against HBV (IC50 range: 10–20 μM). Mechanistic studies showed that compound 12b reduced HBV mRNA levels and HBsAg production with an IC50 of 16.24 μM, approximately 100-fold less potent than RG7834. This reduced potency may be attributed to differences in cellular uptake efficiency or target selectivity.

The RNA stability of HBV and HAV depends on the host non-canonical poly(A) polymerases PAPD5 and PAPD7, while RG7834 can inhibit these enzymes, thereby reducing HBsAg expression. PAPD5 and PAPD7 also function as host mRNA metabolic regulators involved in RNA quality control. Complete inhibition of these polymerases disrupts RNA metabolism and stability, leading to toxicity. Toxicological studies have demonstrated that RG7834 causes mitochondrial and ocular toxicity, which were primary factors in the termination of its clinical development.

In the design of drugs for treating hepatitis, the introduction of fluorine plays a crucial role by leveraging its unique atomic properties—high electronegativity and small atomic radius. For example, HAP-PROTAC and GLP26-PROTACs.At the molecular level, fluorine atoms optimize the charge distribution of drug molecules through electronic effects, thereby enhancing binding affinity to targets such as the hepatitis B virus polymerase and increasing antiviral activity. Simultaneously, fluorine blocks metabolically vulnerable sites (e.g., C–H bonds), significantly improving the metabolic stability of the drug and prolonging its duration of action. From a pharmacokinetic perspective, fluorination increases the lipophilicity of the drug, facilitating its transport across cell membranes, which leads to higher intracellular drug concentrations and sustains effective blood levels for longer periods—allowing for reduced dosing frequency. In summary, by optimizing molecular structure, fluorine serves as a core strategy for enhancing antiviral efficacy, extending therapeutic duration, and reducing side effects, making it highly valuable in modern antiviral drug development.

### 3.2 PROTAC against HCV

The earliest approved therapeutic agents for HCV were inhibitors targeting the viral NS3/4A protease. However, these drugs like telaprevir (VX-950) often lose efficacy due to viral resistance mutations. In August 2019, Professor Priscilla L. Yang’s team published a pioneering study in Nature Communications proposing a novel antiviral strategy based on PROTAC. By designing bifunctional small-molecule degraders, they effectively inhibited HCV while reducing susceptibility to resistance mutations, offering new perspectives for antiviral drug development (De Wispelaere et al., 2019).

The research team utilized telaprevir (VX-950) as the foundation, conjugating it with an E3 ubiquitin ligase ligand to create dual-functional compounds possessing both inhibitory and degradative capabilities (Figure 3). Through structural analysis, they identified the solvent-exposed pyrazine ring of VX-950 as the optimal attachment site and developed three degraders (DGY-03-081, DGY-04-035, and DGY-08-097) by derivatizing this moiety with various linkers connected to cereblon (CRBN), the substrate receptor of the CUL4-RBX1-DDB1-CRBN E3 ubiquitin ligase complex.

Among these, DGY-08-097 demonstrated superior characteristics: it exhibited an IC50 of 247 nM against the NS3/4A protease and efficiently degraded the NS3 protein (DC50 = 50 nM) via the CRBN-dependent ubiquitin-proteasome pathway. Quantitative proteomics analysis confirmed the highly selective degradation of NS3 without affecting other host proteins.

In HCV-infected cell models, DGY-08-097 demonstrated antiviral activity with an IC50 of 748 nM. Its efficacy primarily depended on CRBN-mediated degradation rather than simple protease inhibition. The essential role of this degradation mechanism was confirmed through experiments using CRBN knockout cells and competitive assays. Additionally, MLN4924, an inhibitor of the CRL4 complex, was able to reverse NS3 degradation, further supporting the ubiquitination-dependent nature of this process.

The study also evaluated the degraders’ effectiveness against resistant mutants. For VX-950-resistant mutations V55A and A156S, DGY-08-097 demonstrated superior resistance profiles compared to traditional inhibitors. Its potency against the A156S mutant decreased only threefold, compared to telaprevir’s tenfold reduction. This suggests that DGY-08-097’s lower dependence on binding affinity enables better efficacy against mutation-induced alterations in the binding site.

This study represents the first application of targeted protein degradation technology in the treatment of hepatitis C virus (HCV), demonstrating the feasibility of CRBN-dependent ubiquitin TPD in liver cells. Unlike traditional inhibitors, these degraders eliminate all viral protein functions, including enzymatic activity and structural roles, potentially offering greater therapeutic efficacy.

Recently, it was discovered that telaprevir, which targets the HCV NS3/4A protease, also inhibits the 2A protease of enterovirus D68 (Musharrafieh et al., 2019). This indicates that this PROTAC molecule can degrade the EV-D68 2A protease and exhibit anti-EV-D68 activity. Although these findings demonstrate the antiviral potential of PROTACs only at the cellular level, they strongly confirm that this emerging technology can serve as a promising approach for antiviral drug discovery.

## 4 Advantages and challenges of PROTACs in the development of hepatitis virus therapeutics

Compared to traditional hepatitis medications, PROTACs offer several advantages as antiviral agents for treating viral hepatitis:

1. Catalytic degradation capability: A single PROTAC molecule can dissociate after degrading one target protein and then proceed to degrade additional targets. This catalytic property allows PROTACs to function efficiently at low doses (Bemis et al., 2021), whereas conventional small-molecule drugs require higher concentrations to adequately occupy target sites, increasing the risk of off-target effects.
2. Reduced drug resistance: Viruses readily develop drug-resistant mutations, often necessitating the use of combination therapies involving multiple drugs with distinct mechanisms of action. Targeting viral proteins through degradation rather than inhibition may offer a novel antiviral paradigm that not only helps mitigate the development of resistance but also increases the likelihood of achieving a cure. In the context of hepatitis B virus infection, there is an urgent need for therapeutic agents that target pathways beyond reverse transcriptase inhibition. Furthermore, many scaffold proteins targeted by PROTACs are structurally conserved and less prone to mutation, further minimizing the development of resistance (Gabizon et al., 2020; Li et al., 2022).
3. Enhanced selectivity: Studies demonstrate that PROTACs exhibit degradation selectivity comparable to or even surpassing that of their target protein ligands. By recruiting E3 ligases to specific targets, PROTACs achieve more precise recognition and degradation of designated proteins, thereby improving targeting specificity (Bondeson et al., 2017; Huang et al., 2017). Compared to the degradation of overexpressed endogenous proteins in cancer cells, the selective degradation of exogenous viral proteins is often more readily achievable. Consequently, PROTAC-based strategies represent a promising avenue for the development of novel antiviral agents.
4. Unique pharmacodynamics: Conventional small-molecule inhibitors operate via an “occupancy-driven” mechanism, requiring continuous binding to active sites. In contrast, PROTACs utilize an “event-driven” mode, where sustained catalytic degradation occurs as long as PROTAC molecules are present, resulting in durable effects even at low doses (Buhimschi et al., 2018; Sun et al., 2018).

The PROTAC strategy represents a promising approach for developing anti-hepatitis virus drugs; however, it faces several challenges. First, the limited availability of E3 ligase ligands—primarily CRBN or VHL binders—restricts the optimization of drug activity and resistance profiles (Lee et al., 2022; Sosič et al., 2022). Second, the large molecular weight, poor aqueous solubility, and high polar surface area of PROTACs hinder membrane permeability and penetration of physiological barriers (Han and Sun, 2022). Third, the non-selective expression of E3 ligases across target tissues and normal organs results in off-target biodistribution and potential adverse effects (Sun et al., 2019). This underscores the importance of targeting viral proteins such as HBx and HBcAg for degradation. Additionally, the heterogeneous distribution of E3 ligase types, concentrations, and substrates across subcellular compartments, combined with the dynamic localization of viral proteins during replication, significantly complicates PROTAC design (He et al., 2022). The unique bifunctional architecture of PROTACs, featuring flexible linkers, fundamentally differs from conventional drugs, rendering traditional drug-likeness parameters inadequate and necessitating the accumulation of databases and empirical validation (Liang et al., 2023). While these degraders exhibit potent in vitroactivity against wild-type targets, a major translational hurdle persists: clinical isolates frequently harbor mutations—such as truncations, fusions, or drug-resistant alterations—whose impact on degrader efficacy remains unvalidated.

## 5 Summary and outlook

In summary, while the global epidemic of hepatitis B virus (HBV) remains a significant challenge and current antiviral therapies have multiple limitations, PROTAC technology offers a promising new direction for hepatitis virus treatment. To address these challenges, researchers can adopt a multi-pronged approach: (1) From a molecular design perspective, the selective degradation targets for HBV should be HBx and HBcAg. (2) HBx-PROTACs have demonstrated potential in cell-based studies, significantly reducing HBV replication and antigen levels. However, due to the lack of a fully resolved crystal structure for HBx and the limited discovery of small-molecule ligands, existing HBx-PROTACs are peptide-based, with large molecular weights and poor metabolic stability, making efficient delivery a critical focus in current HBx-PROTAC research. (3) Core protein allosteric modulators (CpAMs) represent one of the most advanced small-molecule direct antiviral strategies in HBV therapy. When CpAMs are designed as the “targeting ligands” of PROTACs, they could theoretically achieve a dual attack on HBV: inducing aberrant capsid assembly on one hand and promoting protein degradation on the other. (4) PROTAC technology, which has been successfully applied in numerous anti-cancer drugs, can be utilized for the targeted degradation of HBV proteins. Photo-responsive PROTACs incorporating nitrobenzyl photocages completely suppress ternary complex formation until activation at 405 nm, achieving over 90% activity recovery and enabling spatiotemporal control to minimize toxicity (Liu et al., 2020). Additionally, microenvironment-responsive designs—such as glutathione-sensitive (disulfide-linked) (Chen et al., 2021), hypoxia-activated (nitroreductase-triggered) (Cheng et al., 2021), and matrix metalloproteinase (MMP)-cleavable variants (Zhang et al., 2021)—allow precise activation under disease-specific conditions (e.g., high redox potential, low oxygen, or protease-rich microenvironments). These strategies reduce off-target toxicity by 60%–80%, paving the way for next-generation anti-hepatitis therapeutics.
